# Supplementary material for: Flavivirus prM interacts with MDA5 and MAVS to inhibit RLR antiviral signaling
Source: Cell Biosci. 2023 Jan 13;13:9. doi: 10.1186/s13578-023-00957-0 (PMC9837762; doi:10.1186/s13578-023-00957-0)
Supplement: Supplementary file 1 — Additional file 1: Fig S1. Flavivirus TBEV proteins inhibit interferon production. (A and B) DAOY cells were mock-infected or infected with TBEV (JL-T75) at a MOI of 1.0, 2.0 and 4.0 or activated by Poly (I:C) (0.5μg) for 12 h, cells were collected and the mRNA level of IFNα and IFNβ were detected by qPCR, GAPDH were using as control. (C and D) HEK293T cells were mock-infected or infected with SeV and TBEV (JL-T75) at a MOI of 1.0 for 12 h, cells were collected and the mRNA level of IFNA and IFNB1 were detected by qPCR, GAPDH were using as control. E The immunoblot analysis of the 11 TBEV proteins. Red boxes indicated the viral proteins of TBEV.(F) Cellular toxicity of TBEV proteins. HEK293T cells were transfected with 150 ng of plasmid for each TBEV expression plasmids, cell viability was analyzed by a luminescent cell viability assay. Cell viability <70% were indicated for red color. Fig S2. TBEV prM protein inhibits the dimerization of IRF3. The Myc-IRF3, HA-MAVS together with TBEV prM or EV plasmids were co-transfected into HEK293T cells, cells were harvested at 30 hpt, and the cell lysates were analyzed by native page. The relative intensity of dimer-IRF3 versus mono-IRF3 was calculated using ImageJ software. Fig S3. The TBEV prM protein is predicted to contain one transmembrane motif. A The transmembrane motifs in the TBEV prM protein were predicted by the TMHMM server, version 2.0. b. B The transmembrane motif is from 130 to 152aa in the TBEV prM protein. Fig S4. Flavivirus TBEV prM colocalize and interact with MDA5 and MAVS. A Flag-prM and RLRs expression plasmids were transfected into HEK293T cells. After 24 h, the cells were fixed and stained by Flag and HA antibodies to analyze the co-location of prM and RLRs. Green, RLR proteins signal; red, TBEV prM signal ; blue, DAPI (the nuclear signal). Intensity profiles of the indicated proteins were analyzed by Image J line scan analysis. Bar, 10 μm. B The expression plasmids of ECFP-prM and EYFP-MDA5/MAVS or emp [file 13578_2023_957_MOESM1_ESM.docx]

**Supplementary Materials for**

**Flavivirus prM interacts with MDA5 and MAVS to inhibit RLR antiviral signaling**

Liyan Sui^1^, Yinghua Zhao^1^, Wenfang Wang^2^, Hongmiao Chi^2^, Tian Tian^2^, Ping Wu^3^, Jinlong Zhang^1^, Yicheng Zhao^1^, Zheng-Kai Wei^4^, Zhijun Hou^3^, Guoqiang Zhou^5^, Guoqing Wang^2^, Zedong Wang^1*^, Quan Liu^1,4*^

^1^ Department of Infectious Diseases, Center of Infectious diseases and Pathogen Biology, Key Laboratory of Organ Regeneration and Transplantation of the Ministry of Education, The First Hospital of Jilin University, State Key Laboratory of Zoonotic Diseases, Changchun, China

^2^ College of Basic Medical Science, Jilin University, Changchun, China

^3^ College of Wildlife and Protected Area, Northeast Forestry University, Harbin, China

^4^ School of Life Sciences and Engineering, Foshan University, Foshan, China

^5^The Biological safety level-3 Laboratory, Changchun Institute of Biological Products Co.，Ltd., Changchun, China

*Correspondence: Zedong Wang (wangzedong@jlu.edu.cn) or Quan Liu (liuquan1973@hotmail.com)

This PDF file includes:

Figures. S1 to S8

Tables S1


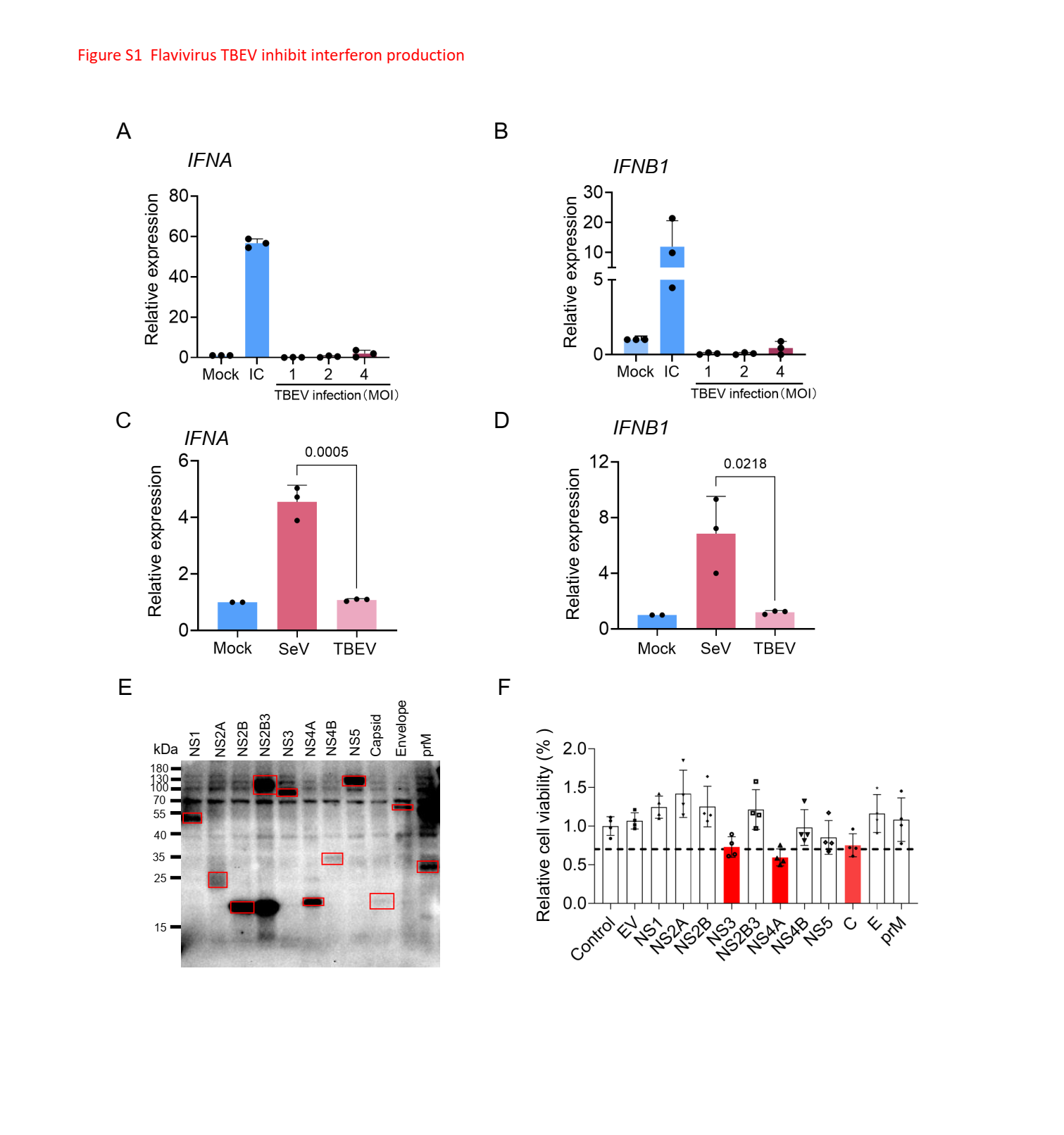


**Fig. S1** Flavivirus TBEV proteins inhibit interferon production**. (A and B)** DAOY cells were mock-infected or infected with TBEV (JL-T75) at a MOI of 1.0, 2.0 and 4.0 or activated by Poly (I:C) (0.5μg) for 12 h, cells were collected and the mRNA level of *IFNα* and *IFNβ* were detected by qPCR, GAPDH were using as control. **(C and D)** HEK293T cells were mock-infected or infected with SeV and TBEV (JL-T75) at a MOI of 1.0 for 12 h, cells were collected and the mRNA level of *IFNA* and *IFNB1* were detected by qPCR, GAPDH were using as control. (**E)** The immunoblot analysis of the 11 TBEV proteins. Red boxes indicated the viral proteins of TBEV.(**F)** Cellular toxicity of TBEV proteins. HEK293T cells were transfected with 150 ng of plasmid for each TBEV expression plasmids, cell viability was analyzed by a luminescent cell viability assay. Cell viability <70% were indicated for red color.


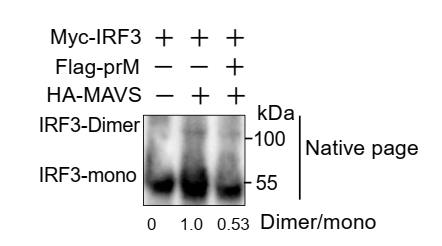


**Fig. S2** TBEV prM protein inhibits the dimerization of IRF3. The Myc-IRF3, HA-MAVS together with TBEV prM or EV plasmids were co-transfected into HEK293T cells, cells were harvested at 30 hpt, and the cell lysates were analyzed by native page. The relative intensity of dimer-IRF3 versus mono-IRF3 was calculated using ImageJ software.


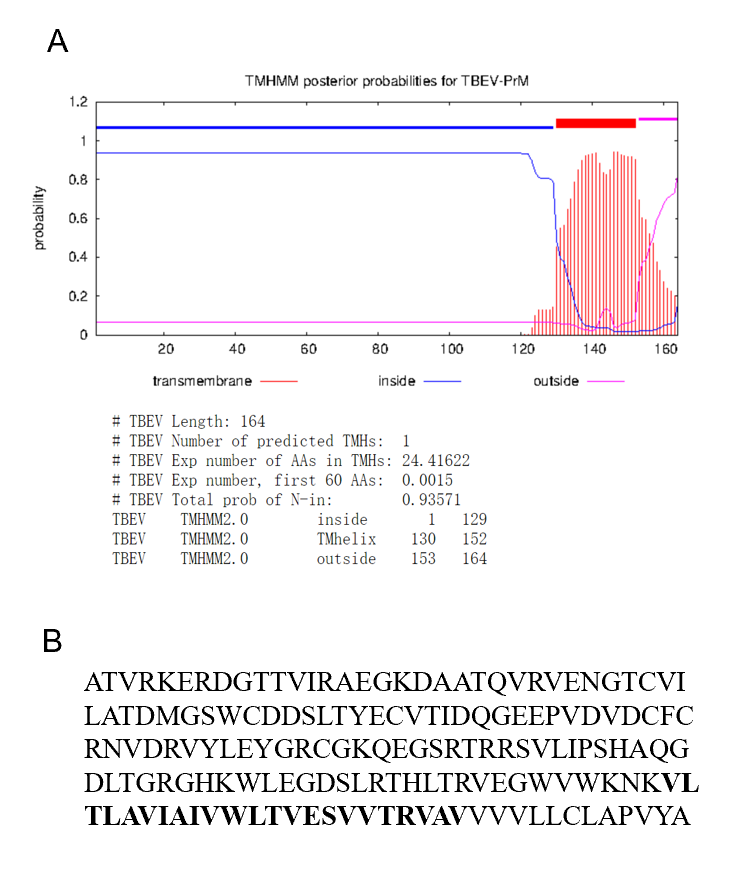


**Fig. S3** The TBEV prM protein is predicted to contain one transmembrane motif. (**A)** The transmembrane motifs in the TBEV prM protein were predicted by the TMHMM server, version 2.0. b. (**B**) The transmembrane motif is from 130 to 152aa (the bold letters) in the TBEV prM protein.


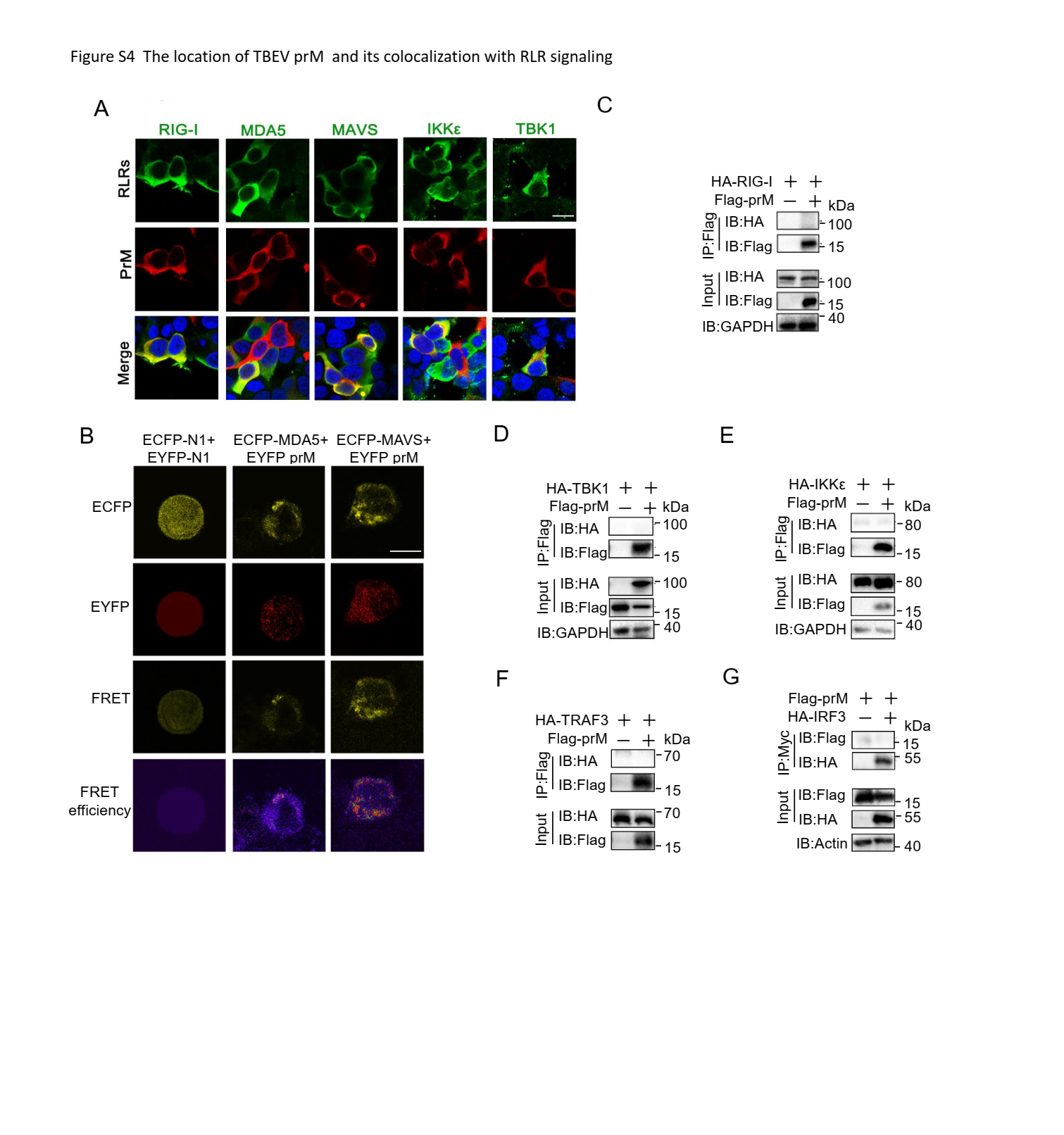


**Fig. S4** Flavivirus TBEV prM colocalize and interact with MDA5 and MAVS. (**A)** Flag-prM and RLRs expression plasmids were transfected into HEK293T cells. After 24 h, the cells were fixed and stained by Flag and HA antibodies to analyze the co-location of prM and RLRs. Green, RLR proteins signal; red, TBEV prM signal ; blue, DAPI (the nuclear signal). Intensity profiles of the indicated proteins were analyzed by Image J line scan analysis. Bar, 10 μm. (**B**) The expression plasmids of ECFP-prM and EYFP-MDA5/MAVS or empty vectors were transfected into HEK293T cells. ECFP-prM provides donor, EYFP-MDA5/MAVS provides acceptor. The FRET and FRET efficiency images were shown at the bottom of the image. Bar, 10 μm. (**C-G)** HEK293T cells were transfected with Flag-prM or EV together with HA-RIG-I (**C**), HA-TBK1 (**D**), HA-IKKε (**E**), HA-TRAF3 (**F**) or Myc-IRF3 (**G**), cells were harvested 30 hpt and the cell lysates were co-immunoprecipitated and analyzed with the indicated antibodies.


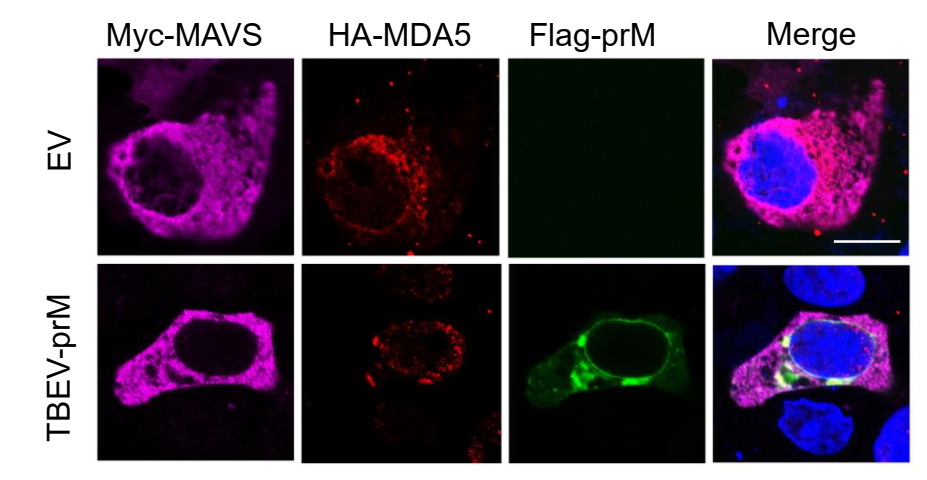


**Fig. S5** HEK293T cells transfected with Myc-MAVS, HA-MDA5 along with EV or Flag-prM were fixed and stained by Flag, HA and Myc antibodies to analyze the co-location of MDA5 and MAVS. Pink: MAVS signal; red, MDA5 signal; green, TBEV prM signal ; blue, DAPI (the nuclear signal). Bar, 10 μm.


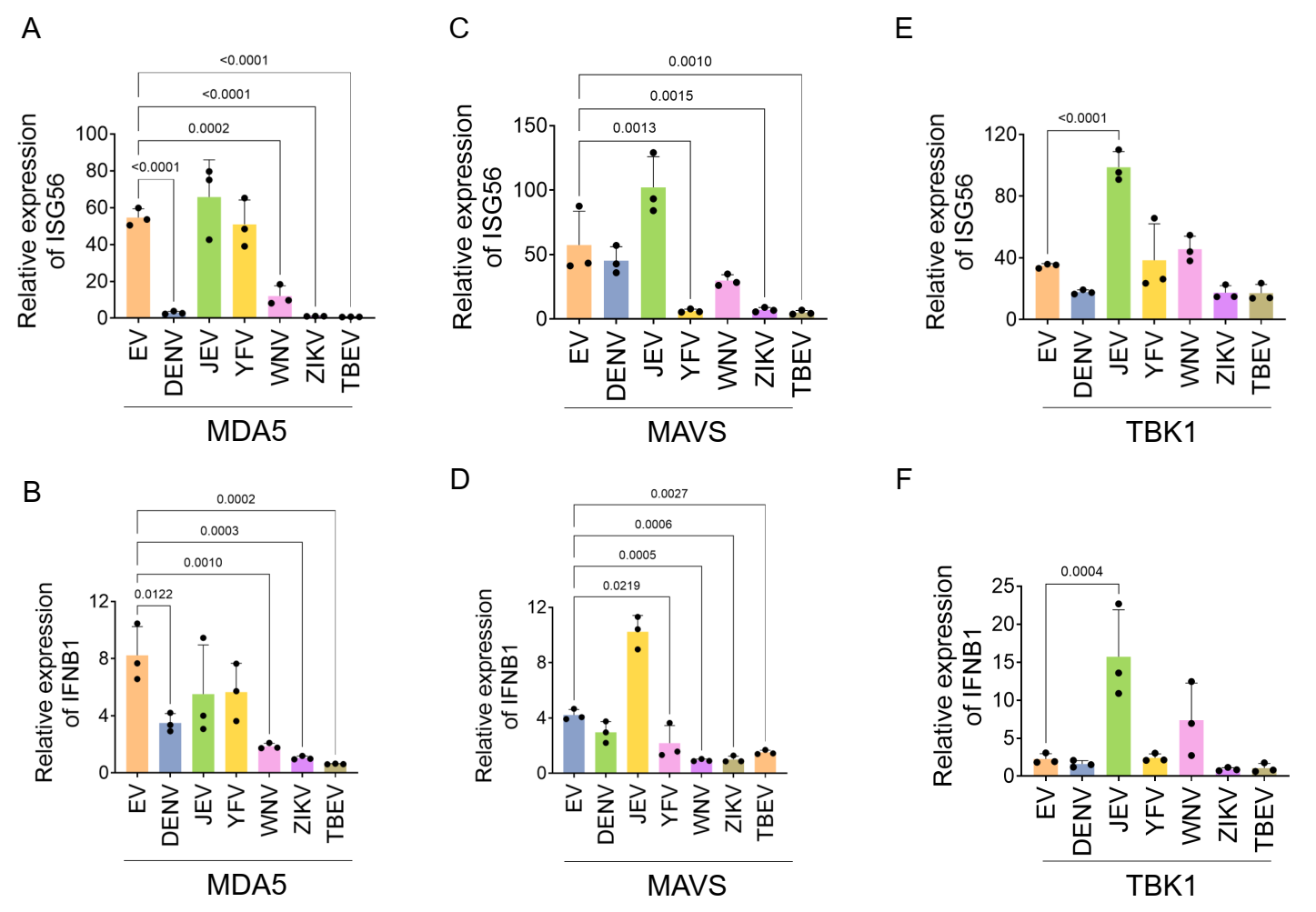


**Fig. S6** EV or flavivirus prM plasmids together with MDA5 (**A**, **B**), MAVS (**C, D**) and TBK1 (**E, F**) were co-transfected into HEK293T cells, the expression of *IFNβ* (**A, C and E**) and *ISG56* (**B, D and F**) were analyzed by qPCR, GAPDH was used as normalizer. Bars represent the mean of three biological replicates and all data are expressed as mean ± SE.


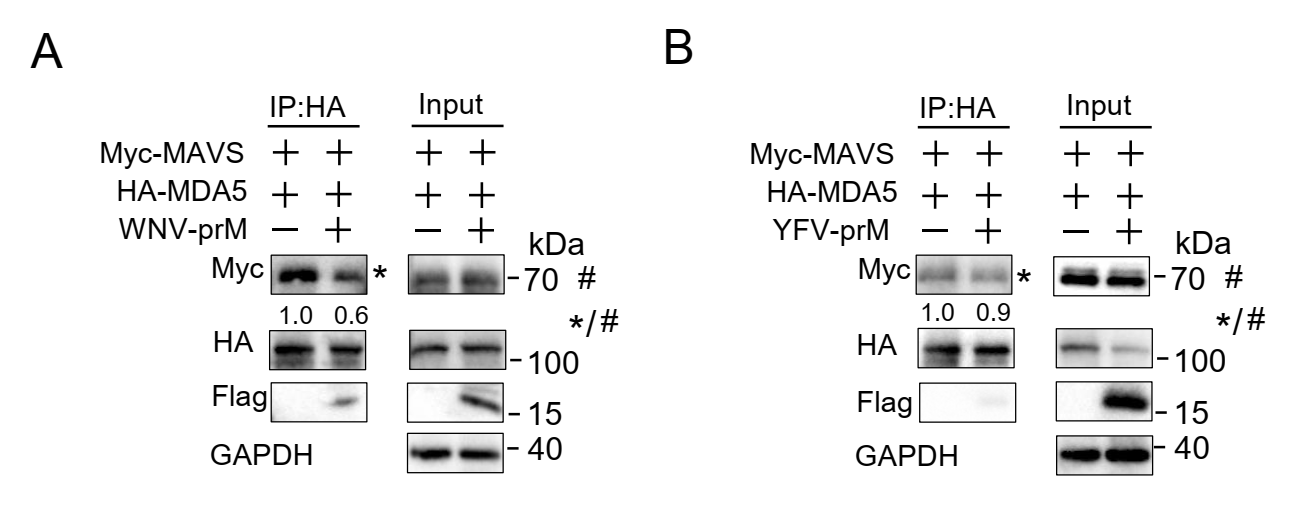


**Fig. S7** WNV prM interferes with the complex of MDA5 and MAVS. Myc-MAVS, HA-MDA5 together with EV or WNV-prM (**A**) and YFV-prM (**B**) were co-transfected into HEK293T cells. After 30 h, cells were harvested and the cell lysates were co-immunoprecipitated with anti-HA antibody. The cell lysates and immunoprecipitants were analyzed by immunoblot using indicated antibodies. The relative band intensity (*/#) of co-immunoprecipitated MAVS was measured using ImageJ software.


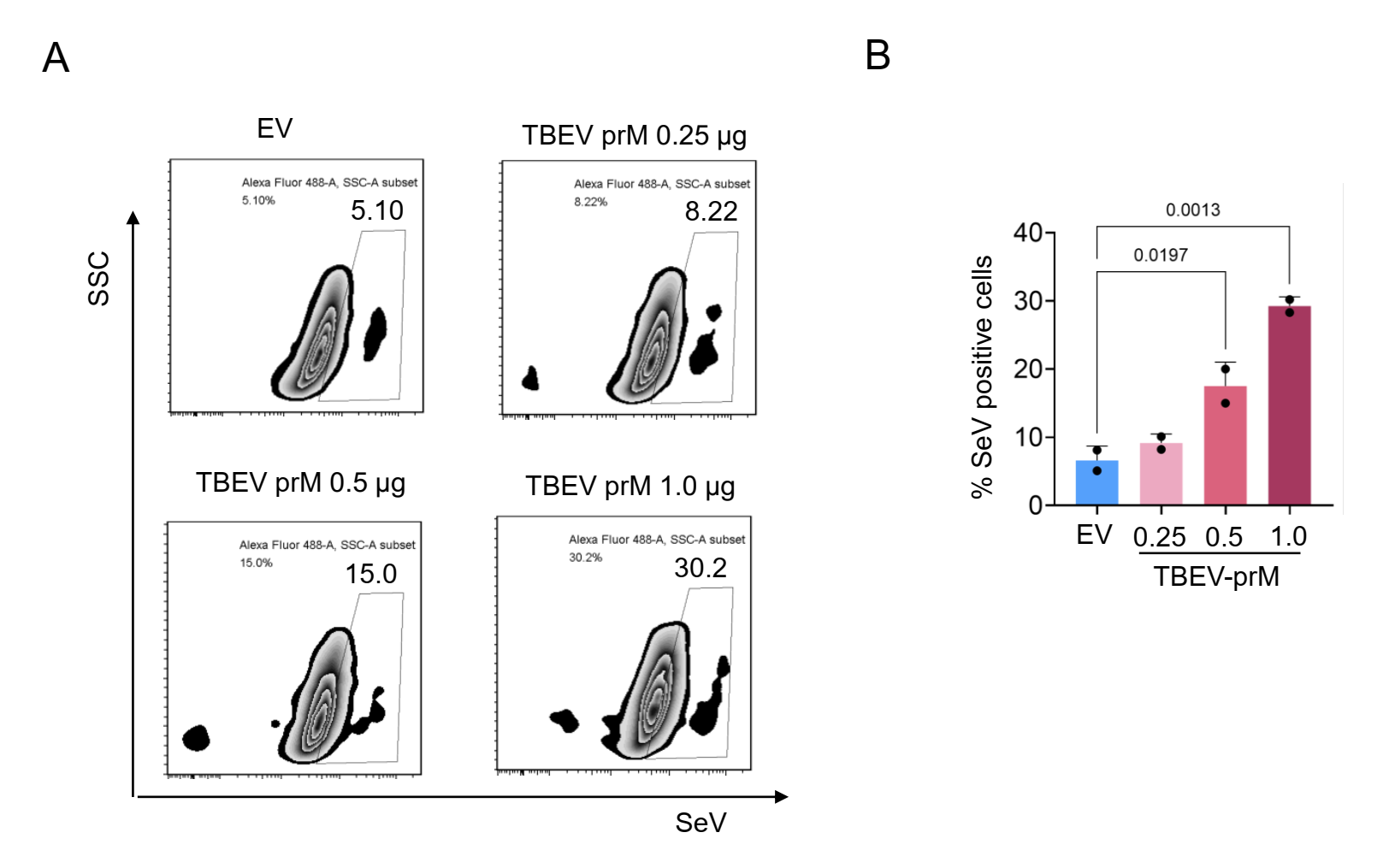


**Fig. S8** The TBEV prM protein facilitate SeV replication. **(A)** EV and TBEV prM plasmids were transfected into HEK293T cells, cells were infected with SeV (MOI 1.0) after 24 h, the replication of SeV was analyzed by flow cytometry analysis. (**B)** Two independent experiments were conducted in A and the data were showed in column graph. *p < 0.05,**p < 0.01.

**Table S1** The prM amino acid similarities of TBEV, DENV-2, JEV, YFV, WNV and ZIKV

|  | DENV2 | ZIKV | YFV | WNV | JEV | TBEV |
| --- | --- | --- | --- | --- | --- | --- |
| DENV2 | *** | 37.5 | 28.6 | 36.3 | 19.0 | 20.2 |
| ZIKV |  | *** | 33.9 | 40.5 | 19.0 | 29.2 |
| YFV |  |  | *** | 33.3 | 14.9 | 26.2 |
| WNV |  |  |  | *** | 38.7 | 25.0 |
| JEV |  |  |  |  | *** | 14.3 |
| TBEV |  |  |  |  |  | *** |
